# 

**Published:** 1888-03-17

**Authors:** 


					n a ?
.PRICE ONE PENNY.
No. tf|7, Vol. III-
March 17th, 1888.
I Registered at the General Pest Office
as a Newspaper.]
0
Per Annum, 6s. 6d.. pott f:ee.
Monthly Farts, 6d.; post free, 7}d.
Ditto per Ann., 7s. ed. post free.
tern Infirmary
gg<y
A Weekly Institutional Journal of
Science, flDebidne, anb [pbtlantvobp^.

				

